# Biofuels and Nanocatalysts: Python Boosting Visualization of Similarities

**DOI:** 10.3390/ma16031175

**Published:** 2023-01-30

**Authors:** Fernando Gomes Souza, Kaushik Pal, Jeffrey Dankwa Ampah, Maria Clara Dantas, Aruzza Araújo, Fabíola Maranhão, Priscila Domingues

**Affiliations:** 1 Biopolymers & Sensors Lab, Instituto de Macromoléculas Professora Eloisa Mano, Centro de Tecnologia-Cidade Universitária, Universidade Federal de Rio de Janeiro, Rio de Janeiro 21941-914, RJ, Brazil; 2 Biopolymers & Sensors Lab, Programa de Engenharia da Nanotecnologia, COPPE, Centro de Tecnologia-Cidade Universitária, Universidade Federal do Rio de Janeiro, Rio de Janeiro 21941-914, RJ, Brazil; 3 University Center for Research and Development (UCRD), Department of Physics, Chandigarh University, Ludhiana–Chandigarh State Hwy, Mohali 140413, Punjab, India; 4 School of Mechanical Engineering, Tianjin University, Tianjin 300072, China; 5 LABPROBIO, Institute of Chemistry, Universidade Federal do Rio Grande do Norte, Natal 59078-970, RN, Brazil

**Keywords:** nanocatalyst, biodiesel, oil, production, reaction, data mining, visualization of similarities method, python, pandas

## Abstract

Among the most relevant themes of modernity, using renewable resources to produce biofuels attracts several countries’ attention, constituting a vital part of the global geopolitical chessboard since humanity’s energy needs will grow faster and faster. Fortunately, advances in personal computing associated with free and open-source software production facilitate this work of prospecting and understanding complex scenarios. Thus, for the development of this work, the keywords “biofuel” and “nanocatalyst” were delivered to the Scopus database, which returned 1071 scientific articles. The titles and abstracts of these papers were saved in Research Information Systems (RIS) format and submitted to automatic analysis via the Visualization of Similarities Method implemented in VOSviewer 1.6.18 software. Then, the data extracted from the VOSviewer were processed by software written in Python, which allowed the use of the network data generated by the Visualization of Similarities Method. Thus, it was possible to establish the relationships for the pair between the nodes of all clusters classified by Link Strength Between Items or Terms (LSBI) or by year. Indeed, other associations should arouse particular interest in the readers. However, here, the option was for a numerical criterion. However, all data are freely available, and stakeholders can infer other specific connections directly. Therefore, this innovative approach allowed inferring that the most recent pairs of terms associate the need to produce biofuels from microorganisms’ oils besides cerium oxide nanoparticles to improve the performance of fuel mixtures by reducing the emission of hydrocarbons (HC) and oxides of nitrogen (NOx).

## 1. Introduction

Biofuel is any material used to generate energy from biomass [1]. Biofuels’ energy source comes from biomass, which stores the sun’s energy as chemical energy [2]. Several different biomass sources include aquatic and terrestrial plants, forest and agricultural residues, vegetable oils, and municipal and industrial waste [3]. The main types of biofuels are biodiesel [4], biogas [5], and bioethanol [6]. Despite the numerous advantages, such as environmental sustainability [7] and the potential to fully or partially replace fossil fuels [8], biofuels carry some disadvantages, such as pollution caused by intensive crops, high water consumption, the loss of biological diversity, and food habitats [9]. There is also a concern that using crops to produce biofuels would increase the price of agricultural food products [10]. Thus, developing more efficient methods for biofuel production is key to the best use of renewable energy sources, providing the desired transition from petroleum-derived fuels to fuels from sustainable sources without the need to increase agricultural areas [11]. For this, using more efficient catalytic systems [12], such as inorganic materials called nanocatalysts (NCs), is an embracing pursuit, thus, distinguishing the NCs as leading players in the nanocatalysis field [13,14,15,16,17,18,19,20].

Nanocatalysis bridges the gap between homogeneous and heterogeneous catalysis, combining both advantages [21]. NCs have a high surface area, increasing the contact between the reactants and the catalyst surface and significantly increasing catalytic activity [22]. Besides that, reactions in the presence of NCs occur under mild operating conditions. As they usually are magnetizable species, NCs are easily separable from the reaction medium due to their insolubility [23]. According to the existing literature [24,25,26], relevant examples of NCs include nanoparticles containing cobalt [27,28,29,30,31], gold [32,33,34], iron [35,36,37,38,39,40], nickel [41,42,43,44], palladium [45,46,47,48,49,50], and platinum [42,51,52,53]. Among the most diverse uses of NCs are biodiesel production [54,55,56,57,58,59,60,61,62,63], dyeing [64,65,66,67,68,69,70,71,72,73], energy storage [74,75,76,77,78,79,80,81,82,83], fuel cells [60,84,85,86,87,88,89,90,91,92], medicine [93,94,95,96,97,98,99,100,101,102], modification of carbon nanotubes [103,104,105,106,107,108,109,110,111,112], solid composite rocket propellants [113,114,115,116,117,118,119,120,121,122], and water purification [123,124,125,126,127,128,129,130,131,132].

The present work deals with biofuel applications, introducing the result of a systematic search for the keywords “biofuels” and “nanocatalysts” in the Scopus database. This search, performed on 31 May 2022, returned 1071 documents. Even in a small context obtained from two keywords (nanocatalysts and biofuels), when put in perspective, the number of documents gathered is equivalent to about twelve copies of the famous book “Harry Potter and the Order of the Phoenix” or about eight copies of Samuel Richardson’s popular novel “Clarisa.” This approximation was performed considering that the analyzed documents possess, on average, ten pages. If a researcher could have eight hours of reading a day and could read about one hundred and thirty-five pages per day, reading those one thousand and seventy-one documents would require seventy-nine full days of work. In other words, the volume of information available in a single search far exceeds the human ability to read and store information, making it impossible to describe the state-of-the-art unless the researcher already has many years of experience in the specific subject. Thus, in a world where people are driven to make decisions faster and faster, processing a large volume of information in a reasonable time is essential, which is perfectly reliable for modern computers. Using these computers and software specially designed for analyzing this textual information is crucial to taking advantage of the vast scientific information available in the scientific repositories. For these reasons, the bibliometric analysis method has become a popular approach within the scientific space for analyzing huge number of documents simultaneously. Based on bibliometric analysis results, one can grasp the knowledge domain of an existing or growing field at a faster rate than reading each document one by one. 

So, today, several research groups use bibliometric tools to track and analyze the evolutionary nuances and research hotspots of their field of study. Among the available studies, the ones developed by researchers from Tianjin University concerning biofuels drew our attention. For instance, the investigation involving the characteristics and perspectives for the use of renewable energy in Africa deserves to be highlighted, where an overview between 1991 and 2021 showed that solar energy, carbon dioxide emissions, and rural electrification are the topics that have been most researched over the years, whereas biofuel consumption is on the rise in the region [133]. In another work, the decarbonization of the road transport industry was studied through the application of low-carbon alcohols (LCA fuels) in internal combustion engines. The study showed that the most relevant topics are combustion, performance, and emission characteristics of LCA-fueled machines [134,135]. A third work studied the decarbonization of the maritime transport industry. The results revealed that liquified natural gas is the most researched alternative shipping fuel, but that methanol, ammonia, and hydrogen are promising fuels for the industry’s decarbonization targets [136].

Thus, here in the present work, the keywords in titles and abstracts were analyzed using the VOSviewer software. This software generates data maps of bibliometric or word networks based on the Visualization of Similarities (VOS) technique proposed by researchers Nees Jan van Eck and Ludo Waltman [137]. The Visualization of Similarities technique is for the literature review analyses [138]. The co-occurrence analysis, shown in Overlay Maps, allows counting the number of articles published simultaneously. The distance between the keywords or nodes can be described as quasi-inversely proportional to the similarity, which is nothing more than the relationship in terms of the keywords’ co-occurrence. More considerable distances indicate weaker relationships, while smaller distances indicate stronger relationships between nodes. Thus, the VOSviewer software uses the Visualization of Similarities metric to build a network of keywords composed of adjectives and nouns, which occur in more than one article. After this calculation, the clusters and nodes are shown on a two-dimensional map [139].

A search on the Scopus database using the words “Visualization of Similarities” and VOSviewer returned eighty-two documents [140,141,142,143,144,145,146,147,148,149,150,151,152,153,154,155,156,157,158,159,160,161,162,163,164,165,166,167,168,169,170,171,172,173,174,175,176,177,178,179,180,181,182,183,184,185,186,187,188,189,190,191,192,193,194,195,196,197,198,199,200,201,202,203,204,205,206,207,208,209,210,211,212,213,214,215,216,217,218,219,220,221] sorted by area. The gathered documents were classified into twenty areas: (1) business, management, and accounting (17.4%), (2) social sciences (16.1%), (3) computer science (14.2%), (4) engineering (10.3%), (5) environmental science (8.4%), (6) medicine (6.5%), (7) energy (5.2%), (8) mathematics (5.2%), (9) decision sciences (3.2%), (10) agricultural and biological sciences (1.9%), (11) arts and humanities (1.9%), (12) economics, econometrics and finance (1.9%), (13) physics and astronomy (1.9%), (14) chemical engineering (1.3%), (15) psychology (1.3%), (16) biochemistry, genetics and molecular biology (0.6%), (17) chemistry (0.6%), (18) Earth and planetary sciences (0.6%), (19) health professions (0.6%), and (20) materials science (0.6%). Therefore, the VOSviewer software is a multidisciplinary tool that makes it easier to understand broad topics.

Despite all the functionality implemented in VOSviewer, direct analysis is limited to the “VOSviewer map file”, which contains nodes, clusters, and link strengths (measured in joint counts of occurrences). A second file called “VOSviewer network file” contains only numerical information, which relates the nodes, pair by pair, with the strength of the connection between them. Therefore, an evolution of the use of the data generated by VOSviewer demands more computational resources implemented in a code used for the first time here in this work. This code, written in Python, defines the node pairs with the highest binding strengths, the node pairs with the most recent annual mean values, and the Euclidean distance between nodes. Those 1071 documents had their titles and abstracts analyzed by clustering techniques via Visualization of Similarities implemented in the VOSviewer software and deepened by data reprocessing using the Pandas Python library. Results referring to the number of publications per year, area of knowledge, and country allowed to draw a global panorama. Besides that, the most recent association of terms among the analyzed documents occurs between “exhaust gas temperature” and “CeO_2_ (Cerium (IV) oxide) nanoparticles-dispersed water–diesel–biodiesel”. Therefore, the collected data point in the direction of the most current scientific efforts to improve the quality of diesel engines, making them less polluting [222].

## 2. Materials and Methods

Worldwide tendencies in research about “biofuels” and “nanocatalysts” were determined by data mining. The names of specific biofuels, such as methanol or ethanol, were not used to avoid the contamination of the research subject with petrochemical ones. All available information was retrieved and analyzed according to the following steps.

First, all articles related to research themes subscribed to the Scopus database were searched, which was chosen because it returned 1071 documents against 35 ones for WoS. Data from papers containing the term “nanotechnology” in the title, abstract, or keywords, using the key TITLE-ABS-KEY (“biofuels”) AND “nanocat*” AND (LIMIT-TO (DOCTYPE, “ar”)) were selected. Then, the gathered information was classified by the number of publications per year, area of knowledge, and country using the Scopus Database tools. The primary data files, including the list of the 1071 scientific papers, are available on GitHub (https://github.com/ftir-mc/Biofuel-nanocatalyst.git (accessed on 31 May 2022)).

Then, the RIS file from Scopus was processed using the VOSviewer software, v. 1.6.18 [139]. The bibliometric classification was made in the “overlay” and “network” modes. Additionally, the files were exported as NET and MAP for overlay and cluster classification, respectively. Data from MAP files were organized by cluster size and total link strength. The top-five nodes in each cluster were selected and plotted. 

**Figure 1:**
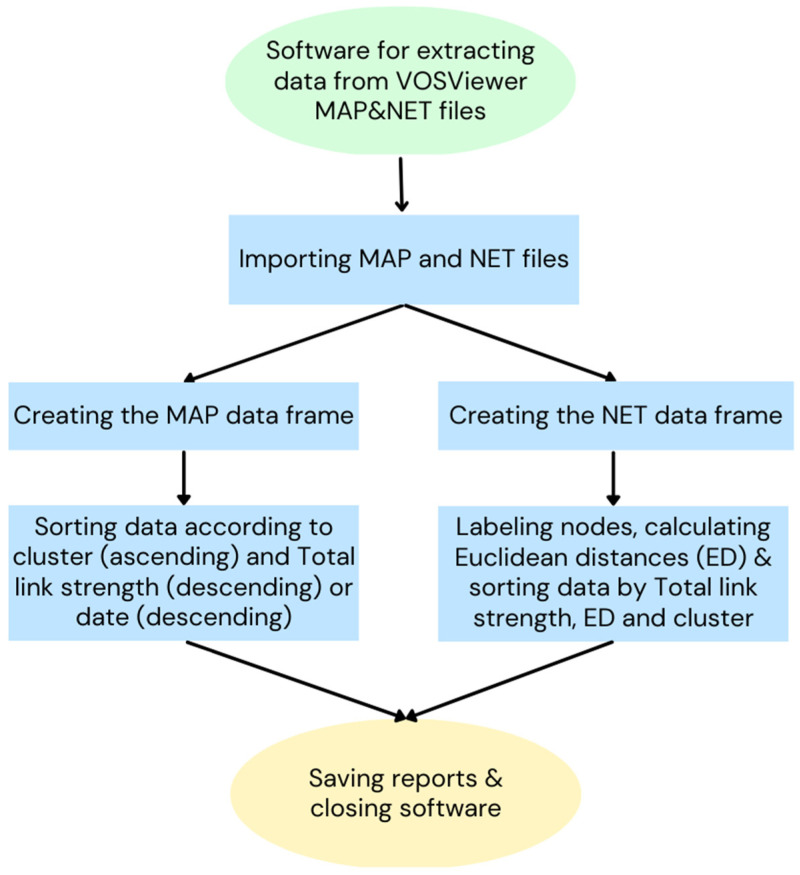
Logical diagram of the developed software.

Finally, a software was written in Python using the Kaleido [223], Matplotlib [224], NumPy [225], Pandas [226], PIL [227], Plotly [228], and Seaborn [229] libraries. Besides those, IPython.display [230], Plotly.express [231], and Statsmodels.stats.multicomp.pairwise_tukeyhsd [232] modules were also used. This software defines the terms (nodes) correlated with each other, pair to pair, initially registered numerically in the first and second columns of the network file generated by VOSviewer. Then, it was possible to identify the nodes with the highest Link Strength Between Items (LSBI) presented in the third column of the VOSviewer network file and the nodes with the most recent annual mean values. In addition, the Euclidean distance between the nodes was calculated. The logical diagram is shown in Figure 1.

## 3. Discussion and Results

To reach the Paris Agreement target of a 1.5–2 °C global warming limit [233], the role of biofuels in substituting liquid fossil fuels cannot be undermined. Considering this, several studies have been conducted over the years to assess the emission reduction potential of replacing conventional fuels with biofuel. Of particular interest, more recent studies have paid keen attention to nanocatalysts as additives for improving the cleaner combustion of both pure fossil fuels and their blends with biofuels for limiting the production of greenhouse gasses [234,235,236,237,238,239,240,241,242,243,244,245,246,247]. Despite the significant contributions to the existing literature on biofuels and nanocatalysts, there is a considerable research gap regarding this field’s evolutionary trends, research hotspots, and characteristics. With this knowledge, the future direction and development of the field can be ascertained, and a well-informed decision for future advancements can be made. So, this work seeks to fill the existing gap and contribute to the existing literature.

The Results Section is presented in subtopics aiming to make the text more accessible to the readers’ understanding. 

### 3.1. Documents per Year

**Figure 2:**
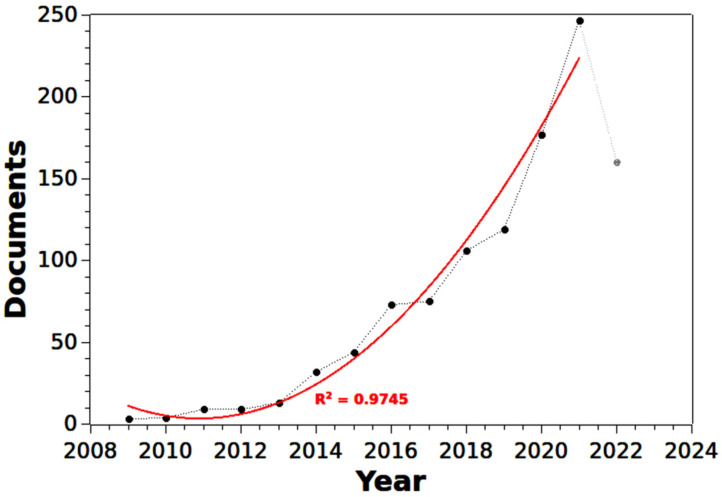
Documents per year (The model does not include 2022).

Figure 2 shows the evolution in the number of published documents on biofuel and nanocatalysts (NCs) in the Scopus database.

The first documents are from 2009. After that date and until 2021, the data trend is described by a polynomial function of order 2, with an R^2^ equal to 0.9745. Using classical mechanics as an analogy, whether Figure 2 presented linear behavior would correspond to the “Uniform Motion”, indicating a continuous and steady increase in interest in each subject over time. In turn, the behavior of the number of publications over the years shown in Figure 2 is similar to the “Uniformly Varied Movement’’. Thus, the “accelerated” behavior of the curve in Figure 2 indicates that interest in the subject increases quickly, driven by the urgency of the brightest human minds for a solution to the anthropogenic environmental devastation that could lead humanity to extinction in a brief time. Another motivator comes from data extracted from the EurObservER Database [248], which shows the price of biofuels in Europe in 2005 at an average value of 48 Euro/MWh. In the following measure, made available in 2010, the average value of biofuels is equal to 59 Euro/MWh, corresponding to an increase of 23%. In 2015, this value stabilized at 58 Euro/MWh, returning to 59 Euro/MWh in 2018 and 2019. These data show that despite the growing demand for biofuels to minimize anthropogenic impacts, these commodity’s value has remained stable, a direct result of the scientific progress registered over the last few years, especially after 2013. Therefore, it is likely that the number of publications will continue to increase rapidly over the next few years.

### 3.2. Documents per Subject Area and Top-10 Main Authors

**Figure 3:**
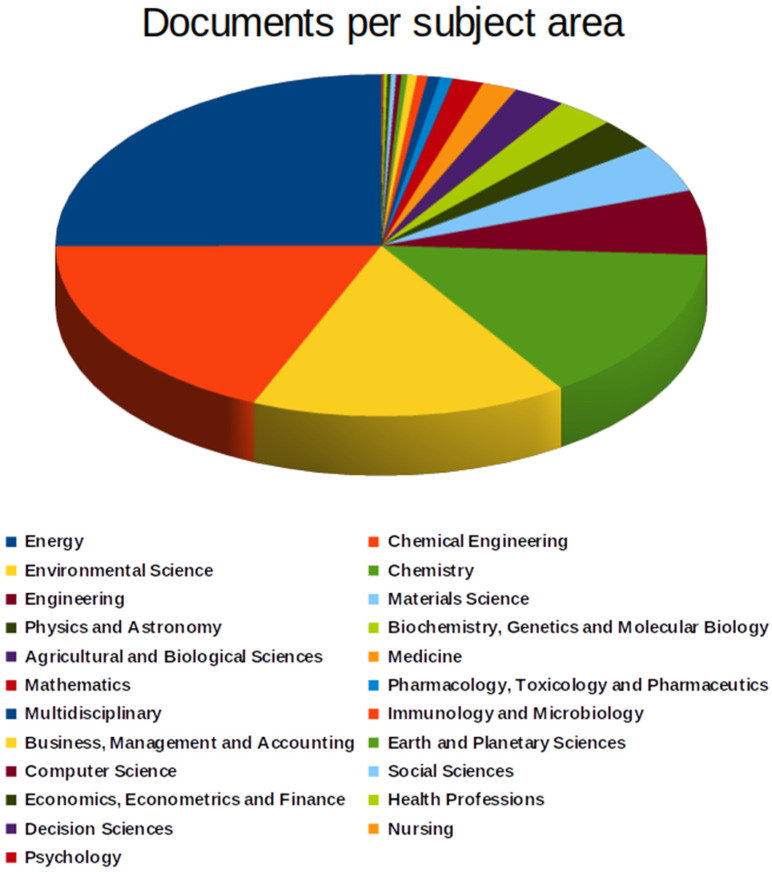
Documents per subject area.

Another exciting classification automatically offered by the Scopus database is the classification by knowledge area, shown in Figure 3.

Among the areas of knowledge, the most remarkable contributions came from energy (579 documents), chemical engineering (427), environmental science (363), chemistry (342), engineering (140), materials science (106), physics and astronomy (69), biochemistry, genetics and molecular biology (66), agricultural and biological sciences (59), and medicine (40). The sum of the number of documents exceeds the total number of articles gathered in this research because each document can be in more than one knowledge area simultaneously.

Regarding journals, the most extensive contributions came from renewable energy (128 documents), bioresource technology (69), fuel (63), energy (30), ACS sustainable chemistry and engineering (24), biomass and bioenergy (22), energy conversion and management (20), green chemistry (18), chemosphere (17), and International Journal of Hydrogen Energy (17).

Besides that, the top 10 scientists working in this area are: Li, H. (Scopus_ID: #35933455300); Yang, S. (Scopus_ID: #7408519878); Haghighi, M. (Scopus_ID: #35237013100); Juan, J.C. (Scopus_ID: #56068042700); Show, P.L. (Scopus_ID: #47861451300); Taufiq-Yap, Y.H. (Scopus_ID: #57194506693); Tavasoli, A. (Scopus_ID: #8937178400); Ahmad, M. (Scopus_ID: #56430353500); Brindhadevi, K. (Scopus_ID: #57200567025); and Ong, H.C. (Scopus_ID: #55310784800). Each contributed 26, 24, 17, 17, 15, 15, 14, 13, 13, and 12 papers, respectively. The specific investigation regarding their contributions was not the aim of this work, but this data could be a fascinating topic for future work.

### 3.3. Documents per Country and Word Cloud

**Figure 4:**
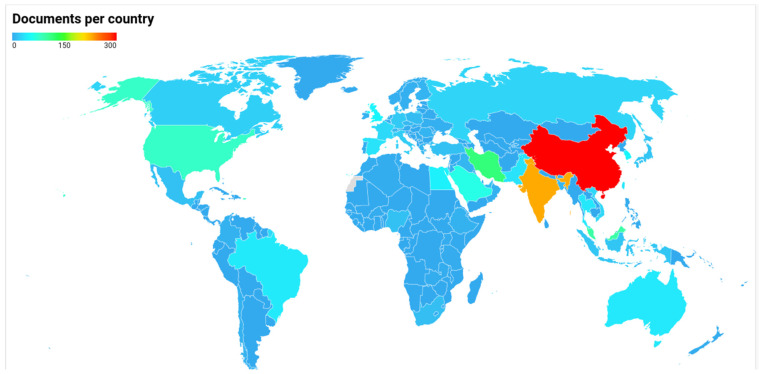
Documents per country.

Figure 4 shows the countries that contributed the most to the theme. Figure 4 was prepared using the DataWrapper online tool, and the original is available at https://datawrapper.dwcdn.net/qMjZN/1/ (accessed on 23 September 2022). The data extracted from the Scopus database have the following classification: China (296 documents), India (229), Iran (115), Malaysia (95), United States (78), Saudi Arabia (62), South Korea (45), United Kingdom (44), Egypt (42), and Brazil (38). These data make it clear that the most prominent players in the field are China and India, countries with huge populations that need all energy sources, including renewable ones. Besides, despite the annual production of biofuels from Iran and Saudi Arabia being equal to zero thousand barrels per day [249,250], these countries are among the most prominent players studying biofuels in the world. Thus, the most extensive global oil producers are preparing for the revolution that will replace the fossil-based energy matrix with a renewable one.

Although these facts about the fundamental areas and the leading players are fascinating, even from a geopolitical point of view, this is not the focus of this work, which is interested in terms and associations of terms in the documents researched.

**Figure 5:**
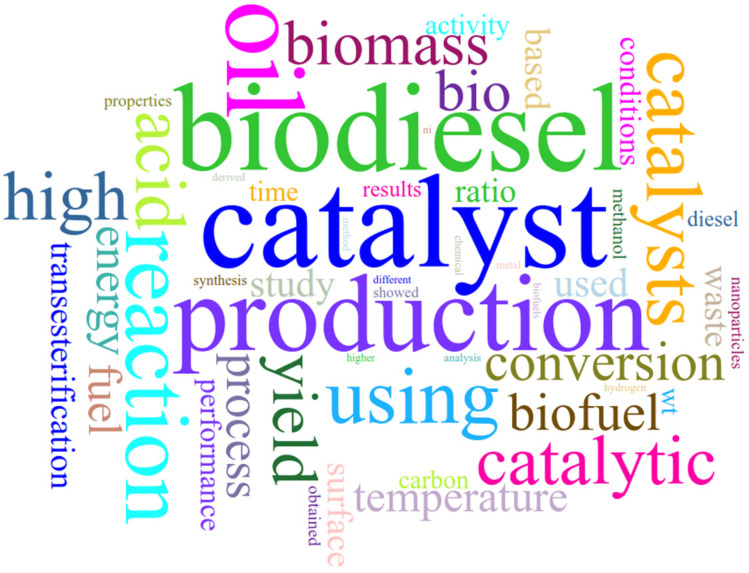
The word cloud from titles and abstracts.

Therefore, the first strategy employed was constructing a word cloud using the words of titles and abstracts. The result is shown in Figure 5.

The visual analysis of Figure 5 allows us to infer that the most frequent terms in the word cloud are “catalyst”, “biodiesel”, and “production”. The present analysis was performed using Voyant Tools, indicating how many times these words are present in the text. More specifically, the most frequent words in the corpus [251] are catalyst (2054 times), biodiesel (1812), oil (1742), production (1483), and reaction (1182).

### 3.4. Visualization of Similarities

All this information presented before is exciting and enriching but of little practical value. Thus, improved tools are essential for understanding the context in which the topic of biofuels and nanocatalysts is inserted and where the technical-scientific focus is heading. The VOSviewer software allows a particular approach based on a method called VOS, meaning “visualization of similarities” [137]. 

**Figure 6:**
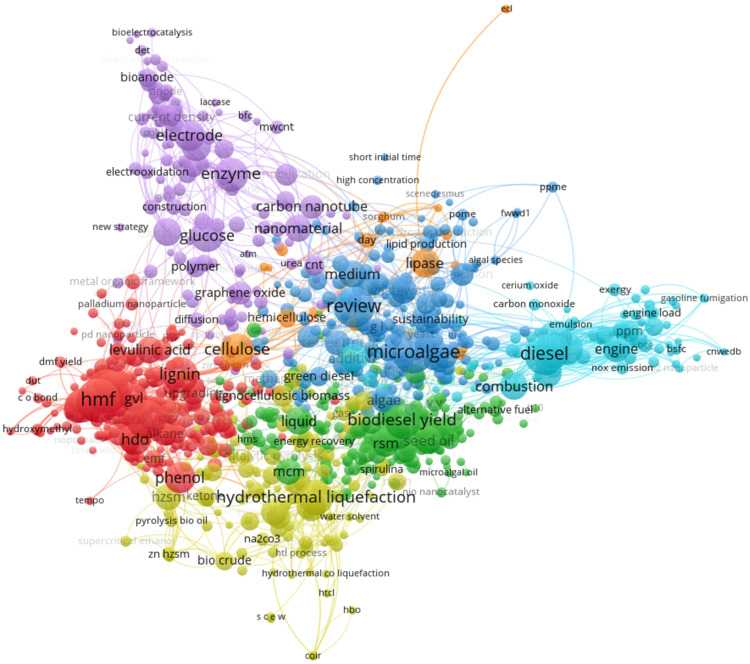
VOS map from titles and abstracts.

**Figure 7:**
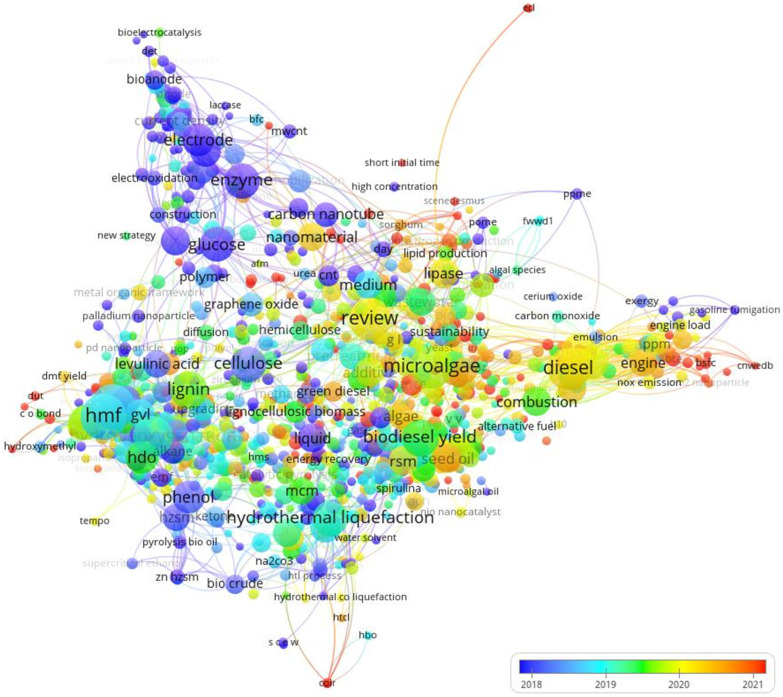
VOS overlay map from titles and abstracts.

Figure 6 and Figure 7 show the maps generated by VOSviewer software using data gathered here. 

The database is available on GitHub, as described in the Methods section. VOSviewer generates a classification by grouping the analyzed texts’ keywords, consisting of a proximity map containing nodes (terms selected by relevance in the number of occurrences) and clusters containing these nodes, as shown in Figure 6. Succinctly, the closer the two terms are, the more significant the correlation between them. The second way of visualizing is via the Overlay map, shown in Figure 7, which presents the same nodes as in the previous case, now sorted by terms’ average years, so that older terms are marked with cold colors while newer terms are warm colors. 

Therefore, Figure 6 shows the existence of seven clusters. In red, the main node of cluster 1 is HMF (5-hydroxymethylfurfural). The main node in cluster 2 in green is “biodiesel yield”. In turn, in cluster 3, in dark blue, the primary node is microalgae. In cluster 4, in yellow, the main node is “hydrothermal liquefaction”. The central node of cluster 5, in purple, is the enzyme. Diesel is the primary node of cluster 6, highlighted in light blue). Finally, cellulose is the central node of cluster 7, highlighted in orange. In turn, Figure 7 shows the evolution of the main theme of this research, where all the most current nodes are in orange-reddish tones, while the older ones are in blue. In general, around the average year of 2018, priority was given to topics involving enzymes, electrodes, and glucose. Between 2019 and 2020, the focus shifted to the yield of biodiesel and microalgae. More recently, between 2020 and 2021, the focus of research shifted to diesel engines, seeking to increase efficiency and reduce the emission of toxic pollutant gasses.

Although functional and visually beautiful, the map representation has several overlays that make analysis difficult.

Thus, the developed code seeks to overcome this disadvantage. The first information provided is regarding the top five nodes of each cluster. So, the top five nodes per cluster are: hmf or 5-hydroxymethylfurfural (cluster 1; Occ. 147), hydrogenation (cluster 1; Occ. 141), hydrodeoxygenation (cluster 1; Occ. 114), lignin (cluster 1; Occ. 95), dmf or 2,5-dimethylfuran (cluster 1; Occ. 81), biodiesel yield (cluster 2; Occ. 97), seed oil (cluster 2; Occ. 60), liquid (cluster 2; Occ. 59), rsm or Response Surface Methodology (cluster 2; Occ. 58), oil molar ratio (cluster 2; Occ. 57), microalgae (cluster 3; Occ. 130), review (cluster 3; Occ. 124), medium (cluster 3; Occ. 57), wastewater (cluster 3; Occ. 53), pretreatment (cluster 3; Occ. 50), hydrothermal liquefaction (cluster 4; Occ. 87), htl or hydrothermal liquefaction (cluster 4; Occ. 66), bio oil yield (cluster 4; Occ. 55), hzsm or protonated zeolite catalysts (cluster 4; Occ. 51), mj kg (cluster 4; Occ. 42), enzyme (cluster 5; Occ. 89), glucose (cluster 5; Occ. 82), electrode (cluster 5; Occ. 76), biofuel cell (cluster 5; Occ. 68), oxidation (cluster 5; Occ. 60), diesel (cluster 6; Occ. 126), blend (cluster 6; Occ. 89), emission (cluster 6; Occ. 87), diesel engine (cluster 6; Occ. 79), combustion (cluster 6; Occ. 60), cellulose (cluster 7; Occ. 85), lipase (cluster 7; Occ. 63), hemicellulose (cluster 7; Occ. 27), day (cluster 7; Occ. 18), viz or videlicet (cluster 7; Occ. 13). 

**Figure 8:**
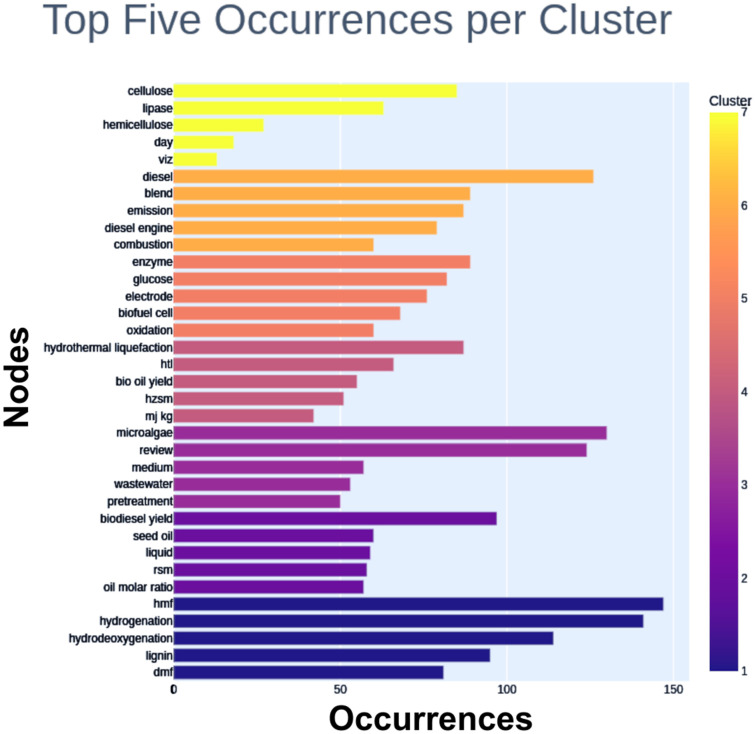
Top five nodes per cluster.

The top five nodes per cluster are also shown in Figure 8.

Regarding the nodes of Cluster 1, shown in Figure 8, second-generation biofuels that use lignocelluloses or celluloses are outstanding alternatives to fossil fuels. Besides, lignocellulosic biomass and carbohydrates are the preferred green, sustainable, and inedible raw materials to prepare various biofuels and valuable chemicals. Furan-based fuels such as 2,5-dimethylfuran (DMF) and 5-hydroxymethylfurfural (HMF) provide a higher energy density than ethanol. DMF is insoluble in water. HMF is a critical intermediate in the DMF synthesis process. DMF is a promising fuel for compression-ignition and spark-ignition engines. These species can improve engine performance, emission, and combustion characteristics compared to other liquid biofuels without modifying the engine structure. Thus, the high energy density, low freezing point, high octane number, high boiling point, high combustion quality, and low pollution emissions make DMF a suitable alternative for commercial gasoline and diesel [252]. Besides being potential biofuels, DMF and HMF are known as intermediates to synthesize other biomaterials and pharmaceuticals [253,254,255,256,257,258], which add value to these molecules.

In turn, regarding the nodes of Cluster 2, the growing concern with the sustainability of several first-generation biofuels is the critical concern of several works that seek the production of biodiesel from non-food crops. This fuel is called second-generation biodiesel, and its main positive points are the consumption of residual oils, the use of abandoned land, and the independence of food crops. Still, the global biofuel production market has not expanded considerably. Among biofuels, biodiesel has the most potential for use as an alternative, biodegradable, renewable, and environmentally friendly fuel. Despite this, production optimization is a vital issue in increasing the scope of this biofuel. For this, the use of residual oils, the selection of inedible oilseed species with high oil yield, and the optimization of processes are fundamental studies [259]. Among the optimization techniques, the response surface methodology stands out due to its advantages, such as the determination of the independent variables’ magnitudes, the ability to model the system mathematically, as well as the time savings and cost reduction due to the smallest number of experiments necessary for the construction of the response surface [260,261,262,263,264,265,266,267,268,269,270,271,272].

As for the nodes of Cluster 3, different wastewater sources such as municipal, agricultural, and industrial contain more significant amounts of organic and inorganic contaminant nutrients released into water bodies without proper treatment, resulting in eutrophication. The main reason for the waste above is the absence of efficient and economical methods for wastewater treatment. However, wastewater is perfect for microalgae growth. These are single-cell photosynthetic organisms capable of growing in wastewater and even sewage. Thus, wastewater treatment with microalgae is advantageous, as it decreases the biochemical oxygen demand (BOD) and the chemical oxygen demand (COD) and removes inorganic nutrients (nitrates and phosphates) from wastewater, in addition to sequestering carbon dioxide via fixation of inorganic carbon from the atmosphere. Despite the incredible versatility of microalgae, wastewater has different compositions and needs to be treated beforehand [273]. Thus, it is often necessary to adjust nutrients and other factors such as temperature, pH, salinity, light intensity, and duration of the microalgae growth process. Another crucial issue is the selection of microalgae species [274,275,276,277,278,279,280,281]. Finally, the microalgae-mediated wastewater treatment can directly produce biofuel (bioelectricity and biohydrogen), besides lipid-rich biomass, essential for biodiesel production [282,283,284].

Concerning Cluster 4, biomass conversion methods consist of biochemical methods such as fermentation and thermochemical methods, which include combustion, pyrolysis, gasification, and liquefaction. Thermochemical liquefaction is an efficient and promising way to convert biomass into solid waste, liquid or bio-crude fuel, and gas. Hydrothermal liquefaction (HTL) is the thermochemical process that treats wet biomass at temperatures between 250 and 350 °C and pressures between 5 and 15 MPa. HTL is conducted in the presence of a solvent, which can be water or alcohol, with or without a catalyst. The catalysts influence the yield and quality of the bio-crude obtained via the HTL process. Various acid or alkaline catalysts can be used. However, they cause corrosion of liquefaction equipment and require additional steps for separation/purification increasing production costs. Thus, replacing conventional catalysts with heterogeneous ecological catalysts is pivotal in improving bio-crude yield and quality in biomass liquefaction [285]. The heterogeneous Ni/HZSM-5 catalyst is hydrothermally stable, improving the pyrolysis bio-oil. Furthermore, the Ni/HZSM-5 catalyst can be reused as heterogeneous solids separated and recovered from the reaction products. In addition, they are disposed of safely [286,287,288,289,290,291,292].

Regarding Cluster 5, obtaining energy from renewable resources is one of humanity’s main goals, and one option for this goal is enzymatic biofuel cells. These devices can convert energy derived from biofuels into electrical energy via the catalytic action of oxidoreductase enzymes. This known technology has been neglected due to its inherent difficulties, albeit the easier and faster development of metallic electrocatalysts for fuel cells. Protein immobilization and stabilization reached the necessary advance only at the end of the 20th century. Due to the incomplete oxidation of biofuels, enzymatic biofuel cells suffer from low energy density. For instance, glucose enzymatic biofuel cells can generate two electrons. However, 24 electrons can be released from glucose, showing that there is still much ground for increasing the efficiency of these devices [293]. The use of enzyme cascades is an alternative to maintaining the high energy densities of biofuel cells and increasing energy density. Enzyme cascades can mimic the metabolic pathways of enzymes to completely oxidize substrates such as ethanol and increase power density by almost ten times compared to a single enzyme ethanol biofuel cell [294,295,296,297,298,299,300,301,302,303,304].

Regarding Cluster 6, the transport sector is the leading consumer of diesel, producing massive emissions in diesel engines. This environmental impact can be minimized or eliminated using blends of diesel with biodiesel or biodiesel alone. Biodiesel is the safest alternative automotive fuel [305], with low particulate and hydrocarbon emissions [306]. However, biodiesel in engines presents challenges due to this biofuel’s low volatility and high viscosity, characteristics restricting fuel spraying, and good air–fuel mixture. Biodiesel–diesel blends need additional studies for their use, and the lack of knowledge of the performance of biodiesel–diesel in diesel engines is the reason for the low use of the blend of these fuels. Some of the limitations of biodiesel as a fuel are its high viscosity [307], high oxygen content [308], and high combustion temperature [309], which increase NOx emissions [310]. The diesel engine design must be modified to use biodiesel without additives, allowing for efficient self-ignition and fuel lubricity, which can be achieved using oxygenated compounds such as ethanol. Several studies discuss diesel-alcohol and diesel–biodiesel–alcohol mixtures. Although biodiesel blend in diesel engines has many advantages, the main disadvantage is low oxidative stability, generating peroxides and hydroperoxides and monomeric, oligomeric, and short-chain compounds formed via rearrangement, fission, and dimerization reactions [311]. Although the IC engine guarantees low fuel consumption and low carbon dioxide emissions, this engine is a source of particulate matter and nitrogen oxide emissions, with unfavorable effects on human health and the environment [311,312,313,314,315,316,317,318,319,320,321,322,323,324]. Therefore, studies on fuel mixtures and new engine designs are essential for expanding the use of biofuels.

Regarding Cluster 7, a trend in the versatile development of biomass decomposition techniques involves cellulase enzymes from multiple domains of bacteria. The enzymatic decomposition of cellulose depends on glycosidic hydrolases and oxidative enzymes. Several organisms secrete cocktails of “free enzymes” that synergistically degrade biomass. Enzymatic action involving three-dimensional (3D) arrangements of proteins and the chemical biology of enzymes are emerging fields. However, the physicochemical recalcitrance of cellulose and chitin limits rapid and economic degradation. Most commercial enzymes are of fungal origin. Bacterial cellulosomes increase the hydrolytic activity of fungal cellulase. Methods for producing cellulosic liquid biofuels by enzymatic hydrolysis have been developed since the end of the 20th Century [293,325]. Advances such as genetic engineering have opened new horizons in this field of study, and several pieces of research have been developed [326,327,328,329,330,331,332,333].

Table 1 shows the principal information extracted from data, sorted by the respective nodes’ highest LSBI values (top) and most recent years (bottom). The results shown in Table 1 are the direct result of the software developed especially for this work, which allows associating the numerical information provided in the network file with the labels, years, and the strength of the links of the files generated by VOSviewer.

Regarding the highest values of Link Strength Between Items or Terms (LSBI), the 2,5-Dimethylfuran (DMF) vs. 5-Hydroxymethylfurfural (HMF) appears twice in Table 1 (see lines 1 and 4), with LSBI values equal to 234 and 115, respectively. The observed repetition is that the terms appear written in abbreviated and complete forms. Something similar occurs in lines 2 and 5, which have the repeated dimethylfuran vs. dmf and htl vs. hydrothermal liquefaction (HTL). Thus, only two sets of pairs should be considered, which are (i) 2,5-Dimethylfuran (DMF) vs. 5-Hydroxymethylfurfural (HMF) and (ii) blend vs. diesel. The following software version should search the corpus looking for abbreviations, thus avoiding repeated terms appearing. Once again, as discussed above, it is clear the immense importance of HMF and DMF as alternative fuels, which can add extra value due to their ability to be used as precursors for several other chemicals. In addition, the other duo, blend and diesel, has relevance due to the continuous process of researching innovations and improvements in IC engines, which are responsible for most of the land transport performed by humans. This research is fundamental for reducing the anthropocentric impact of particulates and carbon dioxide emissions responsible for several environmental imbalances.

**Figure 9:**
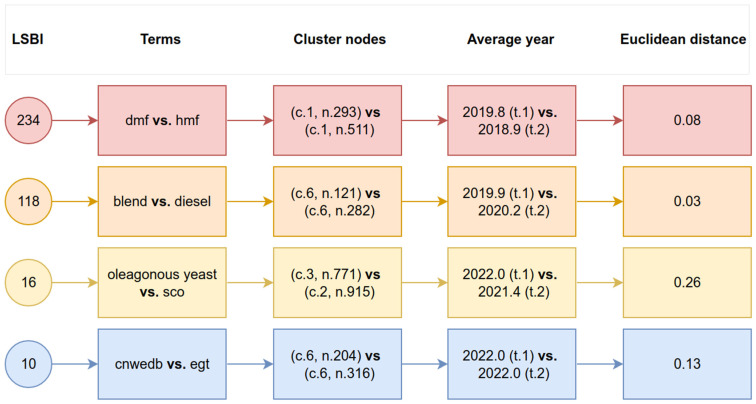
Relevant data from Table 1 sorted by LSBI.

Unfortunately, the use of acronyms and abbreviations by the authors of the papers produces a certain degree of results duplication, which were identified and discarded in the final global analysis. This way, the non-duplicated information from Table 1 is presented as a diagram in Figure 9 to facilitate the discussion.

Concerning the most recently connected terms, shown at the bottom of Table 1, the CeO_2_ nanoparticles-dispersed water–diesel–biodiesel fuel blend (CNWEDB) vs. temperature of the engine exhaust (EGT) appears twice in Table 1, lines 6 and 10, and have LSBI values equivalent to 10 and 5, respectively. Something similar occurs in line 9, which has the repeated terms temperature of the engine exhaust (EGT) vs. temperature of the engine exhaust (EGT). Thus, only three sets of pairs could be considered. However, among the three possible candidates, only two presented higher LSBI values, which are (iii) CeO_2_ nanoparticles-dispersed water–diesel–biodiesel fuel blend (CNWEDB) vs. temperature of the engine exhaust (EGT) and (iv) oleaginous yeast vs. single cell oils (SCO). Their LSBI values are equal to 10 and 16, respectively. Among these same two pairs, the first has values from more recent years [2022.0 (t.1) vs. 2022.0 (t.2)] than the year values presented by the second [2022.0 (t.1) vs. 2021.44 (t.2)].

Regarding the first pair of more modern terms, there is scientific evidence that the oxygen available in biodiesel reduces the carbon monoxide concentration [334] and the IC engine’s hydrocarbon emissions [335]. On the other hand, as a significant disadvantage, biodiesel’s higher oxygen content leads to higher nitrogen oxides (NOx) [336]. Unlike pure biodiesel, NOx emissions can be reduced using water-in-biodiesel fuel emulsions. In addition, some experimental studies investigated the use of cerium (IV) oxide (CeO_2_) nanoparticles as an additive in diesel–biodiesel fuel mixtures and their impact on the thermal and environmental behavior of the CI diesel engine. Hydrocarbon (HC) emissions are reduced by up to 50% with cerium oxide immobilized on amide-functionalized multiwall carbon nanotubes (MWCNT) NCs dispersed in the B20 mixture [337,338,339,340]. The engine in this mixture also produced lower carbon monoxide (CO) emissions than the base fuel. More recently, it has been proven that the presence of CeO_2_ nanoparticles in water–diesel–biodiesel fuel blend increases the engine’s brake thermal efficiency (BTE) by 7.65% over diesel. Additionally, the heat losses were observed at 80% engine load for CNWEDB, indicating a minimum better fuel energy converted to useful work [341].

Finally, about the second pair of more recent terms, yeasts are microbial agents for the efficient production of free fatty acids, fatty alcohols, and alkanes [342]. For instance, the yeasts *Rhodotorula glutinis* and *Rhodosporidium toruloides* can store more than 80% of lipids in their biomass [343]. Microbial oils derived from oleaginous yeasts, fungi, bacteria, and algae are also known as single-cell oils (SCOs) [344]. Oleaginous yeasts can utilize various cheap carbon resources, including agro-industrial wastes such as wheat bran, sugarcane molasses, corn husk, wheat straw, and paper mill waste, making SCO production commercially viable and sustainable [345]. Thus, a series of tailings can be used, reducing the environmental impact of several monocultures and even untreated effluents [346,347,348,349].

Therefore, this work establishes that the use of yeasts for producing fats that later will be transformed into biodiesel and systems based on cerium nanoparticles are critical themes for the scientific and technological developments related to the energetic use of renewable resources.

## 4. Conclusions, Outlooks, and Recommendations

A myriad of scientific documents are produced annually on the most diverse topics. Thus, understanding the paths taken during scientific advances in each area is often challenging, and relevant scientific data remain hidden in these documents. So, developing strategies for understanding advances in topics of interest is crucial for good scientific work. Thus, this work established a new data handling procedure assisted by the Visualization of Similarities Method and Python.

In this study, we analyzed data from over a thousand scientific articles from Scopus. Qualitative and quantitative research tools allowed the mapping of the set of publications on the topic composed of the terms “nanocatalyst” and “biofuel”. The results revealed that the growth in publications was slow between 2009 and 2012. However, after 2013, there was a sharp growth in the number of publications. The growth in the number of publications follows a polynomial function of order 2, with a correlation equal to 0.9872. This rise is related to the increase in energy prices and the understanding that anthropogenic impacts are increasingly devastating to the environment. 

The three central knowledge areas related to this study were energy, chemical engineering, and environmental science. The analysis of the countries’ performance regarding scientific content on the studied subject showed that China contributed the most to research production, followed by India, Iran, Malaysia, the United States, Saudi Arabia, South Korea, the United Kingdom, Egypt, and Brazil. The presence of China and India is a direct result of the population surplus, which is greedy for unlimited energy resources. Iran and Saudi Arabia, among the leading players on the subject, indicate their preparation for the inevitable change in the world’s energy matrix.

The VOS analysis showed the existence of seven clusters. Besides, VOS showed the migration of focal interest over the years, starting from subjects such as enzymes, electrodes, and glucose, evolving to biodiesel yield and microalgae, and finally to diesel engines and the emission of toxic pollutant gas reduction.

The software developed for this study can show the main clusters and their five primary nodes. In addition, the software can list the top five link strengths between terms and the top five most recent linked terms. Unfortunately, the use of acronyms and abbreviations by the authors of the papers produces a certain degree of duplication of results, which were identified and discarded in the final global analysis. So, the next version of the software should search the corpus looking for abbreviations, avoiding repeated terms appearing.

Therefore, two pairs with the highest LSBI values remained among the five pairs: DMF vs. HMF and blend vs. diesel. The first one is related to the ability of these two substances to be used as alternative fuels, which are also precursors of several other chemicals. The accentuated importance of the second pair is due to the continuous search for improving fuel and internal combustion engines.

In turn, among the most recent pairs, two stood out. They are CeO_2_ nanoparticles-dispersed water–diesel–biodiesel fuel blend vs. temperature of the engine exhaust and oleaginous yeast vs. single cell oils. The first pair highlights the search for ways to reduce CO and NOx emissions. The latter can decrease to less than 50% with cerium oxide and B20 blends. The second pair shows a search for microorganisms capable of processing oils and fats, reducing dependence on monocultures and even allowing the use of untreated effluents as precursor environments for biofuel production.

Thus, the concern with energy efficiency and environmental preservation is critical to the scientific and technological developments related to using renewable resources as energy.

## Figures and Tables

**Table 1:** Main information from software containing the top five LSBI and top five most recent linked terms.

**Top Five Link Strength between Terms**			
**Terms (t.1 vs. t.2)**	**LSBI ↓**	**Years (t.1 vs. t.2)**	**E.D.**
1. dmf vs. hmf	234	2019.77 (t.1) vs. 2018.86 (t.2)	0.08
2. dimethylfuran vs. dmf	125	2019.84 (t.1) vs. 2019.77 (t.2)	0.03
3. blend vs. diesel	118	2019.99 (t.1) vs. 2020.16 (t.2)	0.04
4. dimethylfuran vs. hmf	115	2019.84 (t.1) vs. 2018.86 (t.2)	0.11
5. htl vs. hydrothermal liquefaction	112	2018.89 (t.1) vs. 2019.16 (t.2)	0.05
**Top five most recent linked terms**			
**Terms (t.1 vs. t.2)**	**LSBI**	**Years (t.1 vs. t.2) ↓**	**E.D.**
6. cnwedb vs. egt	10	2022.0 (t.1) vs. 2022.0 (t.2)	0.13
7. egt vs. scp	4	2022.0 (t.1) vs. 2021.67 (t.2)	0.69
8. oleaginous yeast vs. sco	16	2022.0 (t.1) vs. 2021.44 (t.2)	0.26
9. egt vs. exhaust gas temperature	4	2022.0 (t.1) vs. 2021.4 (t.2)	0.13
10. cnwedb vs. exhaust gas temperature	5	2022.0 (t.1) vs. 2021.4 (t.2)	0.26

## Data Availability

The primary data files are available on GitHub (https://github.com/ftir-mc/Biofuel-nanocatalyst.git (accessed on 6 December 2022) and https://doi.org/10.32388/XCHU6M (accessed on 6 December 2022).
